# Supplementary material for: Circulating miRNAs as Biomarkers of Obesity and Obesity-Associated Comorbidities in Children and Adolescents: A Systematic Review
Source: Nutrients. 2019 Nov 27;11(12):2890. doi: 10.3390/nu11122890 (PMC6950354; doi:10.3390/nu11122890)
Supplement: Supplementary file 1 [file nutrients-11-02890-s001.pdf]

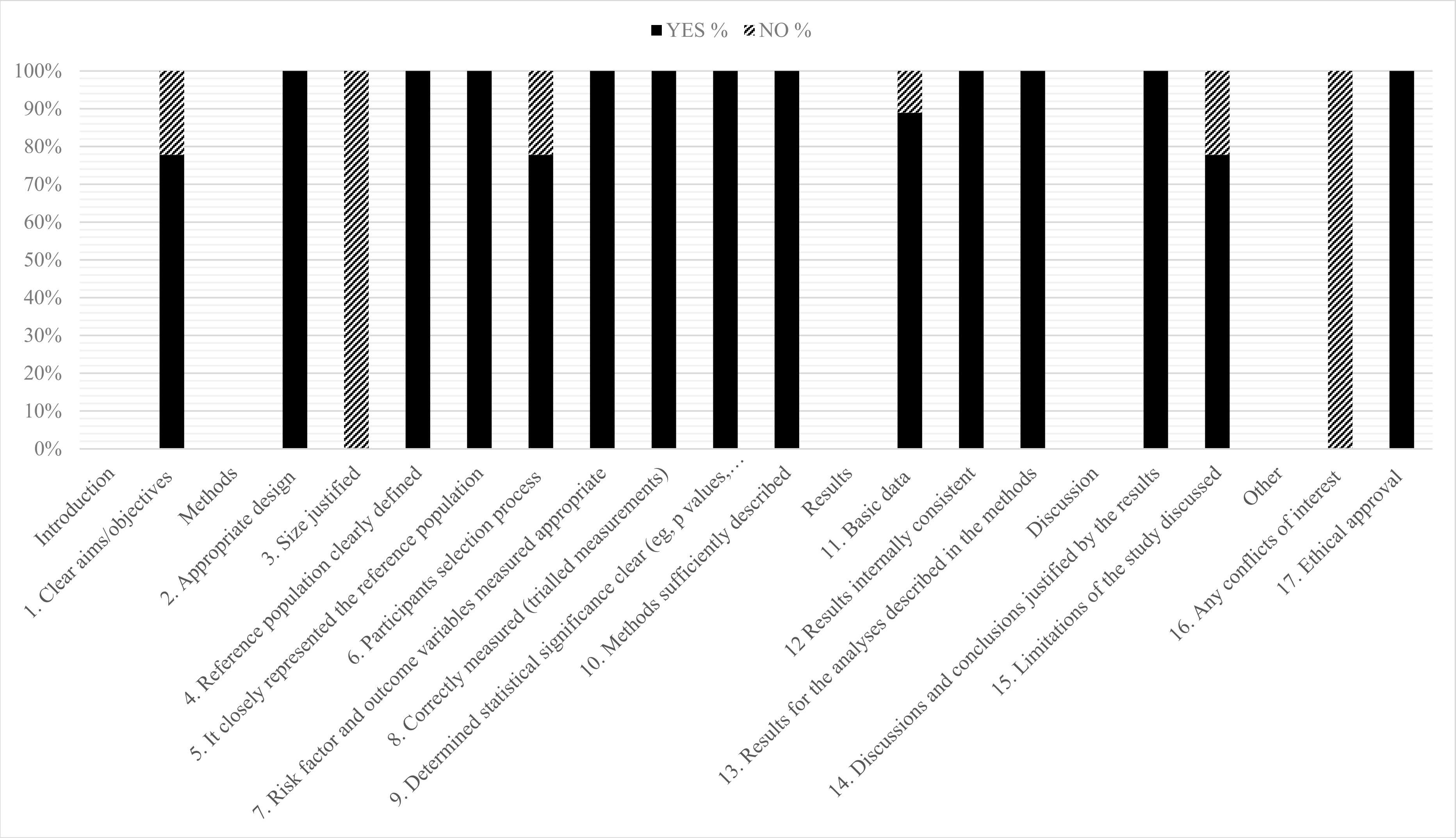

Figure S1: Risk of Bias Assessment and Study Quality

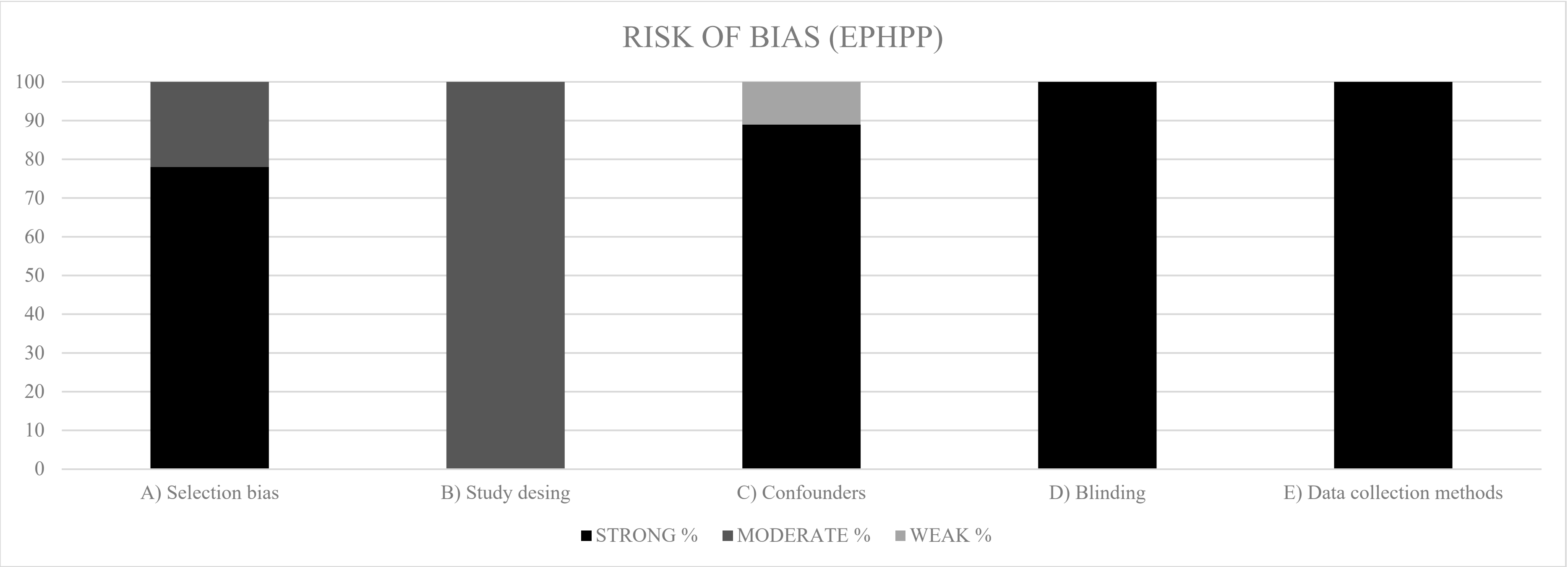

Figure S2: Risk of Bias Assessment and Study Quality (EPHPP; Effective Public Health Practice

Table S1. Differences in miRNA expression between children with obesity and normal-weight children.

**miRNAs**	**Author**	**Effect size (cases vs controls)**			**p**
		**Relative expression level**	**Mean expression level**	**Fold change**	
**miR-222**	Al-rawaf HA et al. 2018	14.5 *vs* 4.5	-	-	<0.001
	Cui et al. 2017	-	-	>6	<0.01
	Prats-Puig et al 2013.	-	41.08±30.59 *vs*. 25.43±17.87	-	0.001
**miR-142-3p**	Al-rawaf HA et al. 2018	12 *vs* 2.5	-	-	<0.001
	Prats-Puig et al 2013.	-	90.31±61.46 *vs*. 32.30±21.29	-	<0.0001
**miR-140-5p**	Al-rawaf HA et al. 2018	13.5 *vs* 4	-	-	<0.001
	Prats-Puig et al 2013.	-	32.66±18.13 *vs*. 23.15±17.50	-	0.001
**miR-143**	Al-rawaf HA et al. 2018	14 *vs* 3.5	-	-	<0.001
	Can et al. 2015	-	30.5 *vs* 115.35	-	0.001
**miR-532-5p**	Al-rawaf HA et al. 2018	8 *vs* 17	-	-	<0.001
	Prats-Puig et al 2013.	-	10.49±7.75 *vs*. 5.49±4.28	-	0.001
**miR-423-5p**	Al-rawaf HA et al. 2018	4 *vs* 14	-	-	<0.001
	Prats-Puig et al 2013.	-	2.16±1.35 *vs*. 1.13±0.77	-	<0.0001
**miR-146a**	Al-rawaf HA et al. 2018	4 *vs* 15	-	-	<0.001
	Cui et al. 2017	-	-	3.8	<0.01
miR-26b	Cui et al. 2017	-	-	3.2	<0.01
miR-26b-5p	Iacomino et al. 2016	-	-	25.37	<0.05
miR-486	Cui et al. 2017	-	-	>6	<0.01
miR-486-5p	Prats-Puig et al 2013.	-	88.75±61.51 *vs*. 40.17±28.44	-	<0.0001
miR-486-3p	Prats-Puig et al 2013.	-	13.92±10.44 *vs*. 7.33±5.53	-	<0.0001
miR-130	Al-rawaf HA et al. 2018	16 *vs* 5	-		<0.001
miR-130a-3p	Ouyang et al. 2017	-	-	1.38	0.018
miR-130b	Prats-Puig et al 2013.	-	24.32±8.46 *vs*. 16.19±9.58	-	<0.0001
miR-520c-3p	Al-rawaf HA et al. 2018	6 *vs* 18	-	-	<0.001
miR-15a	Al-rawaf HA et al. 2018	9 *vs* 23	-	-	<0.001
miR-15b	Cui et al. 2017	-	-	>6	<0.01

miR-146b	Cui et al. 2017	-	-	>6	<0.01
miR-20a	Cui et al. 2017	-	-	3.2	<0.05
miR-197	Cui et al. 2017	-	-	- 4.2	<0.01
miR-301a-3p	Ouyang et al. 2017	-	-	1.33	0.011
miR-199a-3p/199b-3p	Ouyang et al. 2017	-	-	1.5	0.017
miR-191-5p	Ouyang et al. 2017	-	-	1.26	0.018
miR-361-5p	Ouyang et al. 2017	-	-	1.33	0.031
miR-126-3p	Ouyang et al. 2017	-	-	1.26	0.042
Let-7g-5p	Ouyang et al. 2017	-	-	-1.16	0.043
miR-4454	Ouyang et al. 2017	-	-	-2.12	0.043
miR-31-5p	Iacomino et al. 2016	-	-	4.9499	<0.05
miR-2355-5p	Iacomino et al. 2016	-	-	6.5216	<0.05
miR-1231	Iacomino et al. 2016	-	-	-8.7217	<0.05
miR-361-3p	Iacomino et al. 2016	-	-	-4.8918	<0.05
miR-136-5p	Iacomino et al. 2016	-	-	-4.8356	<0.05
miR-320a	Iacomino et al. 2016	-	-	-9.9692	<0.05
miR-206	Iacomino et al. 2016	-	-	-6.0515	<0.05
miR-335	Can et al. 2015	-	2.6 *vs* 11.6	-	<0.001
miR-27	Can et al. 2015	-	77.0 *vs* 124.00	-	0.032
miR-378	Can et al. 2015	-	6.0 *vs* 18.00	-	<0.001
miR-370	Can et al. 2015	-	501.0 *vs* 1687.0	-	0.045
miR-758	Can et al. 2015	-	175.45 *vs* 482.75	-	0.006
miR-221	Prats-Puig et al 2013.	-	8.49±7.01 *vs*. 50.36±45.32	-	<0.0001
miR-28-3p	Prats-Puig et al 2013.	-	5.21±2.80 *vs*. 8.84±4.06	-	<0.0001
miR-125b	Prats-Puig et al 2013.	-	0.48±0.38 *vs*. 0.92±0.88	-	0.001
miR-16-1	Prats-Puig et al 2013.	-	187.04±117.17 *vs*. 113.22±119.48	-	0.001
miR-328	Prats-Puig et al 2013.	-	7.06±4.02 *vs*. 11.44±9.97	-	0.001
miR-363	Prats-Puig et al 2013.	-	6.11±5.27 *vs*. 3.97±4.02	-	0.001
miR-122	Prats-Puig et al 2013.	-	37.44±35.74 *vs*. 23.46±24.83	-	0.001
miR-33a	Carolan et al. 2013	-	-	-	0.001
miR-33b	Carolan et al. 2013	-	-	-	0.017

Table S2. Differences in miRNA expression between children with obesity and insulin resistance and children with obesity and insulin sensitivity

**Differences in miRNA expression (fast).**			
**miRNAs**	**Author**	**Effect size: fold change (cases *vs* controls)**	**p**
miR-122-5p	Masotti et al. 2016	2.82±0.49	0.037
miR-34a-5p		2.41±0.39	0.032
miR-320a		1.55±0.11	0.014
miR-505-3p		3.11±0,65	0.03
miR-26b-5b		1.63±0.17	0.02
miR-146a-5p		1.48±0.09	0.014
miR-148b-3p		1.47±0.18	0.032
miR-342-3p		1.46±0.25	0.05
miR-190a		-3.04±0.39	0.032
miR-200c-3p		-2.78±0.46	0.032
miR-205-5p		-2.60±0.44	0.032
miR-95		-1.72±0.26	0.032
miR-19a-3p		-1.55± 0.21	0.032
miR-660-5p		-1.50± 0.19	0.032
**Differences in miRNA expression after glucose oral tolerance test**			
**miRNAs**	**Author**	**Effect size: fold change (cases *vs* controls)**	**p**
miR-190a	Masotti et al. 2016	2.04±0.77	0.046
miR-200c-3p		3.88±0.82	0.015
miR-95		-3.31±1.35	0.044
miR-30b-5p		-1.82±0.54	0.027
miR-194-5p		2.05±0.53	0.028
miR-885-5p		4.42±1.42	0.044
miR-424-5p		-2.06±0.84	0.044
miR-301a-3p		-2.50±1.07	0.015
miR- 130b-3p		-2.32±0.92	0.046
miR-584-5p		2.45±1.08	0.046
